# Preparation and Application of Conductive Polyaniline-Coated Thermally Expandable Microspheres

**DOI:** 10.3390/polym11010022

**Published:** 2018-12-24

**Authors:** Shou-Zheng Jiao, Zhi-Cheng Sun, Fu-Rong Li, Mei-Jia Yan, Mei-Juan Cao, Dong-Sheng Li, Yan Liu, Lu-Hai Li

**Affiliations:** Beijing Engineering Research Center of Printed Electronics, Beijing Institute of Graphic Communication, Beijing 102600, China; jiaoszzz@163.com (S.-Z.J.); 18810263701@163.com (F.-R.L.); 17810253108@163.com (M.-J.Y.); caomeijuan@bigc.edu.cn (M.-J.C.); Lidongs2378@163.com (D.-S.L.); Liuyan1406806@163.com (Y.L.); liluhai@bigc.edu.cn (L.-H.L.)

**Keywords:** thermally expandable microspheres, suspension polymerization, conductive polymer, ink, screen-printing

## Abstract

The thermally expandable microspheres (TEMs) were prepared through suspension polymerization with acrylonitrile (AN), methyl methacrylate (MMA) and methyl acrylate (MA) as the main monomers. Simultaneously, iso-pentane, n-hexane, iso-octane and other low-boiling hydrocarbons were prepared as blowing agents under two conditions, including high-pressure nitrogen and atmospheric conditions. The above physical foaming microspheres have a core-shell structure and excellent foaming effects. A layer of polyaniline (PANI) was deposited on the surface of the prepared TEMs by emulsion polymerization to obtain conductive and heat-expandable microspheres. Afterwards, the foaming ink was prepared by mixing the conductive TEMs and water-based ink. Finally, a conductive three-dimensional picture was obtained by screen-printing technology. This paper specifically focuses on the effects of particle size, morphology and the thermal expansion properties of the microspheres. The present research methods expect to obtain microspheres with a high foaming ratio, uniform particle size and antistatic properties, which may be applied to physical foaming ink.

## 1. Introduction

In the early 1970s, Dow Chemical Co. first reported the preparation methods of foaming microcapsules in its patents [1], and the research about foaming microcapsule preparation has developed rapidly since then [2,3,4,5,6]. The interior materials of physical foaming microcapsules are generally organic solvents with low boiling points and spherical shells that are thermoplastic polymer coats. When the microcapsules are heated, the heart materials (low boiling point organic solvents) rapidly evaporate with internal pressure production, and the wall materials begin to soften and expand simultaneously. If the thermoplasticity of the microcapsule wall matches the pressure produced by the core material’s vaporization, the microcapsules will show good thermal expansion performance. Under internal pressure, the wall expansion makes the microsphere size increase and density decrease, usually decreasing from 1000 kg per cubic meter to 30 kg per cubic meter [7,8]. Generally speaking, the diameter of the microcapsule increases several times, and the volume increases by dozens or even hundreds of times after expansion. At the same time, the microcapsules after expansion have relative stability without bouncing back after cooling [9,10].

Because of the unique properties, physical foaming microcapsules have been widely applied in the light industry, such as for decorative wallpaper, printing inks [11,12], three-dimensional printing, microfluidics [13], adhesives [14,15] and automotive impact coatings. In many applications of microcapsules, adjusting the surface properties is of a lot of interest for improving the microsphere/matrix interaction [16,17]. Compared with other conductive polymers [18,19,20], polyaniline has been widely studied and applied due to its easy availability of raw materials, simple synthesis process, and chemical and environmental stability [19]. The combination of polyaniline and thermally expandable microspheres (TEMs) could be used in the fields of printed electronics and antistatic materials [20]. By combining functional materials with printing technology, foaming products with large-scale, low-cost and three-dimensional effects could be obtained and used as textile printing foaming or braille printing products [12]. Studies on the preparations and screen-printing applications in flexible substrates of conductive thermally expandable microspheres have significant value for practical application.

At present, suspension polymerization is the most common method used to prepare physical foaming microcapsules [21,22]. However, the properties of microcapsule products would change greatly in different polymerization conditions. This study proposes to prepare foaming microcapsules by the suspension polymerization method under high-pressure nitrogen and atmospheric air conditions. Meanwhile, several factors influencing microcapsule foaming properties will be investigated. Next, the polyaniline (PANI)-coated thermally expandable microspheres will be successfully prepared by in situ deposition from the solution. Finally, a kind of novel microcapsule with special core-shell structures and physical foaming-conductive ink will be prepared based on the research of structure and properties.

## 2. Materials and Methods

### 2.1. Materials and Experiment

Unless otherwise noted, the following reagents were obtained from commercial suppliers and were used without further purification: Acrylonitrile (AN), 99 wt %; methyl methacrylate (MMA), 99 wt %; azodiisobutyronitrile (AIBN), 98 wt %; methyl acrylate (MA), 95 wt %; 1,4-butanediol dimethyl acrylic (BDDMA), 99 wt%; MgCl_2_·6H_2_O, 95 wt %; n-hexane, 99 wt %; sodium hydroxide, 99 wt %; sodium chloride, 99 wt %; HCl, 37 wt %; cyclohexane, 99 wt %; sodium dodecyl sulfate, 95 wt %; aniline, 99 wt %; ammonium persulfate, 99 wt %.

### 2.2. Preparation of TEMs

To prepare oil phase: The monomer acrylonitrile (14 g), methyl methacrylate (4 g), methacrylate (2 g), initiator azodiisobutyronitrile (0.43 g), crosslinking agent (0.04 g) and foaming agent n-hexane (8.77 g) were added into a flask and mixed with magnetic stirring.

To prepare aqueous phase: NaOH (2.5 g) and distilled water (45 g) were added into a flask and mixed fully for use. MgCl_2_·6H_2_O (6 g) and distilled water (45 g) were added into another flask and mixed fully for use. When both were dissolved completely, the NaOH solution and some sodium dodecyl sulfate were added into a three-necked flask with mechanical agitation for some time. Afterwards, MgCl_2_·6H_2_O solution was added at a certain speed with a funnel and stirred with higher speed to form the relatively stable magnesium hydroxide particles dispersion. Finally, sodium chloride, sodium nitrite and anhydrous ethanol were added into the flask and stirred well to get the water phase of the reaction. The water phase and oil phase were mixed and emulsified with a homogenizer, which made the oil phase fully spread in the water phase.

### 2.3. Preparation of Microcapsules under Different Methods

Preparation of microcapsules under atmospheric air pressure: The uniform suspension was transferred into a boiling three-necked flask with a water bath at 65 °C and reacted for 15–20 h under mechanical agitation at 150–400 rpm. Next, the product was cooled to room temperature and preliminary microcapsules were obtained.

Preparation of microcapsules by high-pressure nitrogen protection: The uniform suspension was carried out in an autoclave and nitrogen was added for 5 min by a nitrogen bottle. When the pressure inside the kettle was maintained between 0.4–0.5 MPa, the reaction was started with a water bath at 65 °C and stirring at a constant rate for 15–20 h. The aqueous phase and the oil phase were dispersed into droplets (about 10 μm) under strong agitation. As the temperature increased, the initiator decomposed to form a radical, which initiated radical polymerization of the monomer. As a result, a polymer film was formed on the surface of the oil droplets. As the polymerization progressed, chain transfer and termination led to the gradual depletion of the monomer, and the thickness of the capsule was continuously increased. After the reaction stopped, the temperature of the pressure kettle was cooled to the room temperature and the initial microcapsules were obtained.

### 2.4. Post-Processing 

The suspending agent was removed from the microsphere particles by adding diluted hydrochloric acid dropwise while stirring, acidifying to adjust pH to 3–4. The residual product was filtered and dried after washing with water repeatedly.

### 2.5. Polyaniline Coating and Ink Preparation

TEMs (6 g) were dispersed homogeneously in 30 mL demonized water. Then aqueous solution of 0.113 g ammonium persulfate was added into the water phase and stirred for 30 min. After thoroughly mixing, 10 mL of an aqueous solution of 0.10 g aniline was slowly added dropwise and the mixture was stirred at room temperature for 22–24 h. Afterwards, the reaction solution was filtered and washed with 1 mol/L of hydrochloric acid. Finally, the filter cake was dried at 50 °C for 5 h to obtain the product.

**Figure 1:**
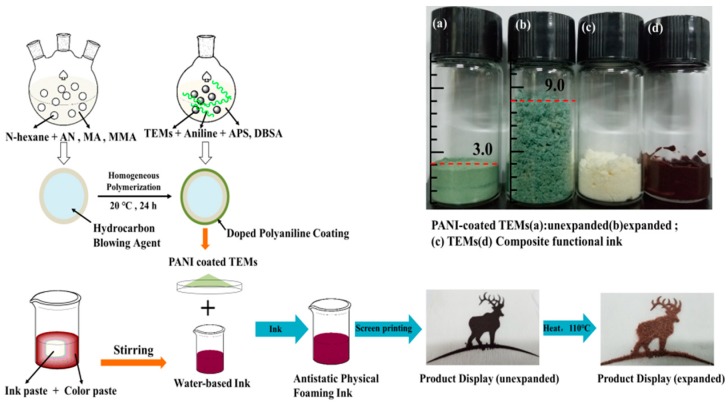
Synthesis of polyaniline-coated TEMs and ink preparation process.

The preparation process of TEMs and the ink are shown in Figure 1. The aromatic ink was prepared in the proportion of printed protoplasmic (50%), water-based acrylic resin (25%), water (5%), paste (9%), TEMs (10%), foaming agents (0.2%) and flatting agent (0.8%). Afterwards, different coated papers, such as aluminum foil paper, non-woven fabric and kraft paper, were used as substrates to transfer the ink. 

### 2.6. Characterization and Instruments

The core-shells of the thermally expandable microcapsules were verified by a Fourier transform infrared test (Nicolet 6700). The thermal performance parameters of the foaming microspheres were analyzed by Thermogravimetric analyzer (TG, NETZCSH TG209F3, NETZCSH Technology Company, Bavaria, Germany), including starting weightlessness temperature, complete volatilization temperature, etc. A DSC (Differential thermal analyzer) curve was obtained through DSC measurement 214 of the German NETZCSH Technology Company (Bavaria, Germany), and the glass transition temperature of the polymer shells was analyzed. A thermal expansion curve was obtained using the thermal expansion measurement instrument (NETZCSH DIL 420 PC, NETZCSH Technology Company, Bavaria, Germany). The precise microsphere foaming temperature (T_start_), maximum foaming temperature (T_max_) and expansion rate (dL/d_0_) were investigated by analyzing the thermal expansion curve. The average size and particle size dispersion (PDI, PDI = (D90 − D10)/D50, D10, D50, and D90 are microcapsule particle diameters of 10%, 50%, and 90%) of the microspheres were measured by the MICROTRAC S3500 laser particle size analyzer (Microtrac Inc, Largo, FL, USA). The surface morphologies of the microspheres were viewed by a scanning electron microscope (HITACHI, SU8020, Tokyo, Japan) and laser confocal microscope. The powder resistivity of PANI-coated TEMs was tested by a high resistance tester (ST2255, Suzhou Jingge, Suzhou, China).

## 3. Results and Discussion

### 3.1. Microcapsule Core-Shell Analysis

**Figure 2:**
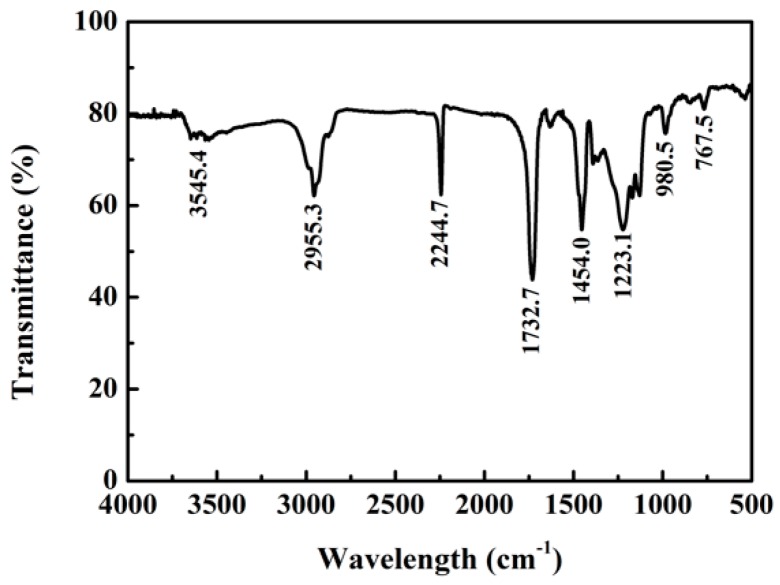
IR spectra of microspheres.

As shown in the infrared spectra of Figure 2, the hydroxyl group in the carboxylic acid of which the peak shape is wide and blunt at 3545.4 cm^−1^ suggested that the polymer formed the intermolecular association by the hydroxyl of carboxylic acid. 2955.3 cm^−1^ is the stretching vibration absorption peak of the core material of isopentane (CH_2_–CH_2_). 2244.7 cm^−1^ is the apparent characteristic absorption peak of –C≡N. 1732.7 cm^−1^ is the characteristic absorption peak of –C=O, whose peak shape is strong and sharp. Through the above analysis, it can be seen that the carboxyl, methyl, ester base and cyano groups contained by the shells of the microspheres are consistent with the characteristic functional groups of synthetic monomers of acrylonitrile, methyl methacrylate and acrylic acid methyl ester. This confirms that the thecal sac polymer is composed of three kinds of monomers from the angle of structure.

### 3.2. Effects of Microcapsule Particle Size on Swelling Property

**Figure 3:**
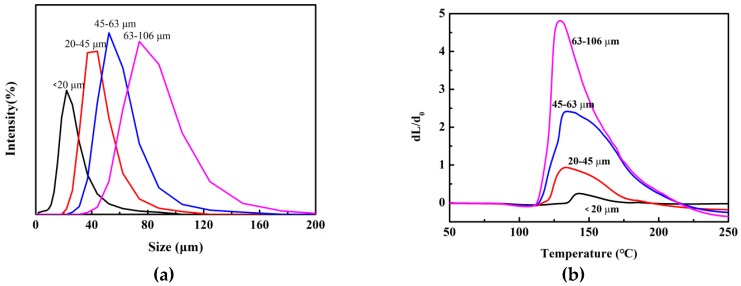
Performance analysis of TEMs with different particle sizes: (**a**) particle size distribution; (**b**) expansion performance.

When the prepared samples were passed through standard screens of 106 μm, 63 μm, 45 μm and 20 μm in turn, four samples of different particle sizes were obtained. The particle size distribution and the morphology of the samples are shown in Figure 3a. It was found by calculation that the particle size dispersions of the above four samples were 0.99, 0.65, 0.62, 0.63 respectively, indicating that the size distribution was narrow of the microspheres (less than 1). Then, the above microsphere samples were tested for their thermal expansion properties, and the thermal expansion curves are shown in Figure 3b. The results show that the different sizes of microsphere expansion ratios had obvious differences, and the expansion ratios of the sample which was passed through 20 µm standard screens were lowest, with a higher starting foaming temperature and steady temperature. As the particle size increased, the initial foaming temperature and the maximum foaming temperature of the microspheres had a tendency to reduce. In addition, the shell thicknesses of microcapsules with different particle sizes were almost the same. The larger particle size of the microcapsules and the more core materials coated in the microspheres resulted in the higher expansion ratio. On the other hand, the smaller microcapsule particle size and a small amount of foaming agent during gasification of internal pressure was not enough to make the wall material expand, causing the foaming effect to be poor. In short, a reasonable choice of different particle size ranges of foaming microspheres will be of great significance for the application of foaming ink.

### 3.3. Investigation of Preparation Methods on the Properties of Expansion

**Figure 4:**
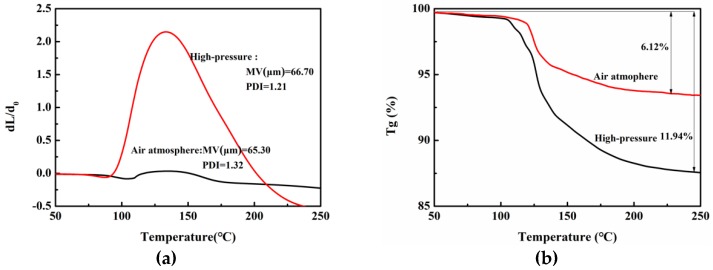
Thermal performance analysis of microspheres under high-pressure nitrogen and atmospheric air conditions: (**a**) Dilatometer (DIL) cures; (**b**) thermogravimetric (TG) cures.

The thermally expandable microcapsules coated with isopentane were prepared in a three-necked flask and autoclave, with average particle sizes (MV) of 65.30–66.70 μm. As shown in Figure 4, the embedding rate of the microspheres under atmospheric air was 6.12% and the expansion ratio was 0.03. The embedding rate of the microspheres under high pressure nitrogen protection was 11.94% and the expansion ratio was 2.14. Isopentane was used as a blowing agent, and it is a low temperature blowing agent because of its boiling point at 30 °C. The reaction temperature was 65 °C, which was much higher than the boiling point of isopentane. Therefore, as the reaction heated up, isopentane evaporated rapidly under the condition of atmospheric pressure reaction, leading to a decrease in the coating rate of core material. Contrastively, the saturated vapor pressure of isopentane increased under the condition of high-pressure reaction, so isopentane existed in its liquid form and participated in the nucleation of the microcapsule. Consequently, when choosing a core material with a low boiling point as the foaming agent, the reaction must be carried out under high pressure in order to ensure that the foaming agent exists as liquid and can be wrapped further.

### 3.4. Effects of Blowing Agent on the Expansion Performance of Microspheres

**Figure 5:**
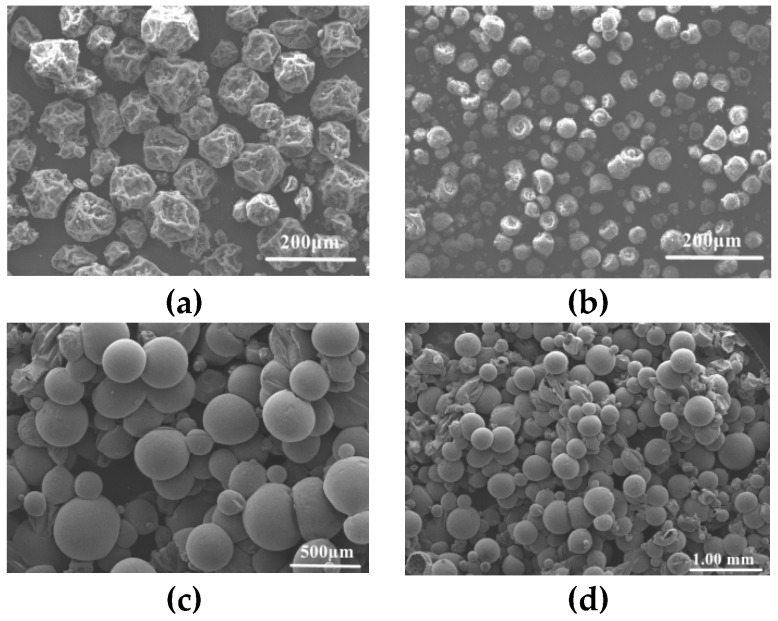
SEM of the microspheres with octane and isooctane as foaming agents: (**a**,**b**) unexpanded; (**c**,**d**) expanded.

As seen from Figure 5, it was found that the particle size distributions of the prepared microspheres are similar when coating different types of foaming agents. As shown in Table 1, the study found the initial foaming temperature of the microcapsules was also lower with low boiling point foaming agents (such as isopentane and n-hexane). The microcapsules have a wide foaming temperature range because the initial foaming temperature is far below the glass transition temperature. Therefore, the microcapsule expansion ratio will also increase with the temperature of the heating mode raised slowly. When the foaming agents with high boiling points (such as n-octane) are used, the microcapsules have a higher initial foaming temperature. The problem is that when the alkane with high boiling point coated in the microcapsule has not vaporized, the outer polymer shell begins to decompose, which results in a lower expansion ratio of microcapsules with high boiling point alkanes. The research on the type and amount of foaming agent shows that for the thermally expandable microcapsules, isopentane, n-hexane and iso-octane are suitable foaming agents.

### 3.5. Effects of Polyaniline Coating on the Properties of Microcapsules

**Figure 6:**
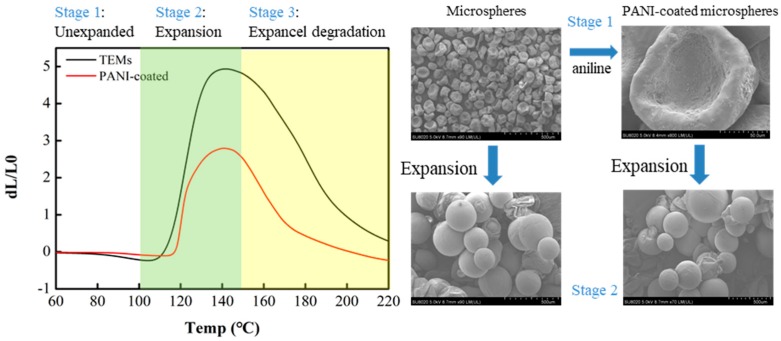
Dilatometer (DIL) curves of uncoated TEMs and polyaniline (PANI)-coated TEMs.

Emulsion polymerization can obtain a larger molecular weight, and a lower amount of oxidizing agent is used in the polymerization process, which can be coated well on the surface of TEMs. The invention has the advantages that the heat of polymerization is effectively dispersed in the water phase, local overheating is avoided, the viscosity of the system is small, and the properties in terms of solubility, thermal stability and crystal morphology are significantly better than solution polymerization [23]. The study of the expansion properties between TEMs and PANI-coated TEMs shows that the expansion properties of microcapsules coated by the conductive polymer are lower than those of uncoated TEMs. An example of this can be seen in Figure 6, in which the expansion ratio of the microspheres before and after immobilization is shown by a DIL. There are several important temperature points obtained, including TEMs’ T_start_ (106.5 °C), PANI-coated TEMs’ T_start_ (116.4 °C), TEMs’ T_max_ (140.4 °C) and PANI -coated TEMs’ T_max_ (140.7 °C). The expansion temperature of the PANI-coated TEMs is higher than the TEMs, showing that the PANI surface-coating acts as a heat-insulator to the TEMs. Furthermore, the maximum expansion ratio (dL/d_0_) of the TEMs is 4.9, and the maximum expansion ratio (dL/d_0_) of the PANI-coated TEMs is 2.8. The expansion ratio of TEMs is much higher than that of PANI-coated TEMs because the volume fraction of microcapsules in PANI-coated TEMs is reduced compared to the same volume of TEMs. In addition, the powder resistivity of PANI-coated TEMs was found to be 493.5 Ω∙cm by a high resistance tester, which indicated PANI-coated TEMs had a certain antistatic property.

### 3.6. Screen-Printing Application of Conductive Thermally Expandable Microspheres in Flexible Substrate 

**Figure 7:**
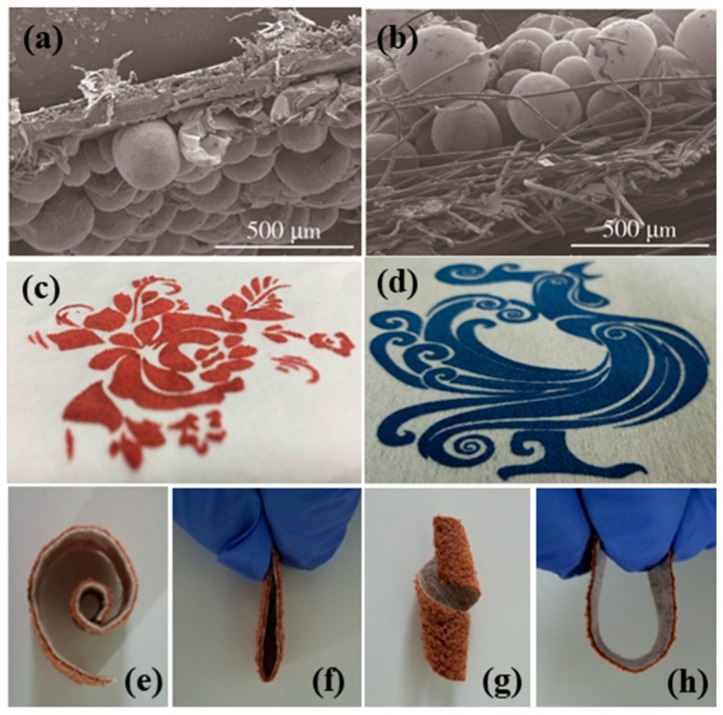
The SEM images of PANI-coated TEMs on different substrate surfaces: (**a**) coated paper, (**b**) non-woven fabric. Screen printing products on different substrates: (**c**) coated paper, (**d**) non-woven fabric. Screen printing products bending performance display: (**e**) curling, (**f**) folding, (**g**) twisting, (**h**) bending.

Screen printing, as a traditional printing method, can be widely applied to different types of substrate surfaces. Figure 7 shows the product pictures and microscopic cross-sectional images of PANI-coated microcapsule inks printed on coated paper and non-woven fabric surfaces. Since the acrylic film-forming resin is added as a binder in the TEMs ink, the TEMs adhere well to the surface of the substrate after being thermally expanded. The pattern of the microcapsule ink can be customized by screen printing. Simultaneously, the pattern after foaming also has the effect of blocking, and plays the role of wallpaper decoration and heat insulation. In addition, due to the coating of PANI, the microcapsule ink is quoted on the surface of clothes, which is not only beautiful but also antistatic. The printing of microcapsule ink can be bended, curled or shaped into other deformations, and the microcapsule inks can still adhere tightly to the surface of the substrate, which proves the possibility of its flexible substrate application.

## 4. Conclusions

In short, the preparation reaction of TEMs embedded in low boiling-point solvent had to be carried out under high pressure conditions in order to ensure that the foaming agent is liquid and wrapped. The types of foaming agents had an important effect on the expansion of microspheres, and the suitable foaming agents for the thermally expandable microcapsules were isopentane, n-hexane and iso-octane. When isopentane was used as a low boiling point foaming agent, the prepared reaction would happen under a certain pressure, ensuring that the isopentane was wrapped and the microcapsule could be obtained. The particle size of the unexpanded TEMs is positively correlated with the expansion properties. Extraordinarily, the polyaniline-coated TEMs still had good swelling and antistatic properties, with powder resistance of up to 493.5 Ω·cm. Finally, the inked TEMs could be applied to different substrates through the screen-printing method, and the flexibly printed products with three-dimensional effects and good adhesive properties were obtained.

## Figures and Tables

**Table 1:** Expandable properties of the microspheres with the different foaming agents.

Foaming Agent	MV (µm)	PDI	Boiling Point/°C	T_start_/°C	T_max_/°C	dL/d_0_
isopentane	66.70	1.21	30	89.50	133.48	2.16
normal hexane	56.23	0.92	68.7	116.76	144.77	2.69
cyclohexane	60.72	0.89	80.7	162.87	176.50	0.09
isooctane	55.78	1.28	99.2	130.39	144.06	2.23
methyl cyclohexane	70.23	0.89	100	141.47	187.33	0.29
normal octane	67.25	0.78	125.6	160.03	198.36	0.16

## References

[1] Morehouse D.S., Tetreault R.J. (1971). Expansible Thermoplastic Polymer Particles Containing Volatile Fluid Foaming Agent and Method of Foaming the Same. U.S. Patent.

[2] Garner J.L., Tiffany P.A. (1979). Method for Expanding Microspheres and Expandable Composition. U.S. Patent.

[3] Melber G.E., Oswald W.A., Wolinski L.E. (1988). Composition and Process for Drying and Expanding Microspheres. U.S. Patent.

[4] Wu H.S., Sun F., Dimonie V.L. (1998). Expandable Hollow Particles. U.S. Patent.

[5] Svedberg L.O., Hovland G., Holmlund T. (2007). Easier Way of Expanding Thermally Expandable Microspheres is Provided Requiring Small Equipment and Reducing Transport Costs of Expanded Microspheres. U.S. Patent.

[6] Svedberg L., Ajdén P. (2016). Method and a Device for Preparation of Expanded Microspheres. U.S. Patent.

[7] Fredlund J. (2011). Synthesis of Thermo Expandable Microspheres. Master’s Thesis.

[8] Hou Z.S., Kan C.Y. (2014). Preparation and properties of thermally expandable polymeric microspheres. Chin. Chem. Lett..

[9] Jonson M., Nordin O., Kron A.L., Malmström E. (2010). Thermally expandable microspheres with excellent expansion characteristics at high temperature. J. Appl. Polym. Sci..

[10] Fujino M., Taniguchi T., Kawaguchi Y. (2013). Mathematical models and numerical simulations of a thermally expandable microballoon for plastic foaming. Chem. Eng. Sci..

[11] Safajou-Jahan Khanemlou M., Abbasi F., Salami-Kalajahi M. (2016). Synthesis and characterization of thermally expandable PMMA-based microcapsules with different cross-linking density. Colloid Polym. Sci..

[12] Urbas R., Elesini U.S. (2015). Color differences and perceptive properties of prints made with microcapsules. J. Graph. Eng. Des..

[13] Jeong J.W., McCall J.G., Shin G., Zhang Y.Y., Al-Hasani R., Kim M., Li S., Sim J.Y., Jang K., Shi Y. (2015). Wireless optofluidic systems for programmable in vivo pharmacology and optogenetics. Cell..

[14] Banea M.D., da Silva L.F.M., Carbas R.J.C., Campilhoc R.D.S.G. (2014). Mechanical and thermal characterization of a structural polyurethane adhesive modified with thermally expandable particles. Int. J. Adhes. Adhes..

[15] Banea M.D., Da Silva L.F.M., Carbas R.J.C. (2015). Debonding on command of adhesive joints for the automotive industry. Int. J. Adhes. Adhes..

[16] Jonsson M., Nyström D., Nordin O., Malmström E. (2009). Surface modification of thermally expandable microspheres by grafting poly (glycidyl methacrylate) using ARGET ATRP. Eur. Polym. J..

[17] Lu Y., Broughton J., Winfield P. (2016). Surface modification of thermally expandable microspheres for enhanced performance of disbondable adhesive. Int. J. Adhes. Adhes..

[18] Cingil H.E., Balmer J.A., Armes S.P., Bain P.S. (2010). Conducting polymer-coated thermally expandable microspheres. Polym. Chem..

[19] Wang H.L., Romero R.J., Mattes B.R. (2000). Effect of processing conditions on the properties of high molecular weight conductive polyaniline fiber. J. Polym. Sci..

[20] Chen S.Y., Sun Z.C., Li L.H., Xiao Y.H., Yu Y.M. (2017). Preparation and characterization of conducting polymer-coated thermally expandable microspheres. Chin. Chem. Lett..

[21] Jonsson M., Nordin O., Malmström E. (2011). Increased onset temperature of expansion in thermally expandable microspheres through combination of crosslinking agents. J. Appl. Polym. Sci..

[22] Jonsson M., Nordin O., Malmström E., Hammer C. (2006). Suspension polymerization of thermally expandable core/shell particles. Polymer.

[23] Subrahmanya S., Rudolf H. (2005). Spectroelectrochemical investigations of soluble polyaniline synthesized via new inverse emulsion pathway. Chem. Mater..

